# Activation-Induced Cytidine Deaminase Initiates Immunoglobulin Gene Conversion and Hypermutation by a Common Intermediate

**DOI:** 10.1371/journal.pbio.0020179

**Published:** 2004-07-13

**Authors:** Hiroshi Arakawa, Huseyin Saribasak, Jean-Marie Buerstedde

**Affiliations:** **1**GSF–National Research Center for Environment and Health, Institute for Molecular Radiobiology, Neuherberg-MunichGermany

## Abstract

Depending on the species and the lymphoid organ, activation-induced cytidine deaminase (AID) expression triggers diversification of the rearranged immunoglobulin (Ig) genes by pseudo V (ψV) gene- templated gene conversion or somatic hypermutation. To investigate how AID can alternatively induce recombination or hypermutation, ψV gene deletions were introduced into the rearranged light chain locus of the DT40 B-cell line. We show that the stepwise removal of the ψV donors not only reduces and eventually abolishes Ig gene conversion, but also activates AID-dependent Ig hypermutation. This strongly supports a model in which AID induces a common modification in the rearranged V(D)J segment, leading to a conversion tract in the presence of nearby donor sequences and to a point mutation in their absence.

## Abstract 2

Activation induced cytidine deaminase (AID) is required for antibody diversification, a process that varies in different species. By removing one diversification pathway in chickens, AID induces an alternative method of diversification

## **Introduction**

Immunoglobulin (Ig) genes are further diversified after V(D)J rearrangement by gene conversion, hypermutation, or a combination of the two. Surprisingly, even closely related species employ different strategies: mice and humans use exclusively hypermutation (Milstein and Rada 1995), whereas rabbits, cows, and pigs use mainly gene conversion (Butler 1998). The balance between the two phenomena can also shift during differentiation: for example, chicken B-cells first develop their Ig repertoire by gene conversion in the bursa (Reynaud et al. 1987; Arakawa and Buerstedde 2004) and later fine tune it by hypermutation in splenic germinal centers (Arakawa et al. 1996).

All three B-cell specific activities of Ig repertoire formation—gene conversion (Arakawa et al. 2002), hypermutation, and isotype switch recombination (Muramatsu et al. 2000; Revy et al. 2000)—require expression of the *activation-induced cytidine deaminase (AID)* gene. Whereas it was initially proposed that AID is an mRNA editing enzyme (Muramatsu et al. 1999), more recent studies indicate that AID directly modifies DNA by deamination of cytosine to uracil (Di Noia and Neuberger 2002). However, the cytosine deamination activity must be further regulated, because only differences in the type, the location, or the processing of the AID-induced DNA modification can explain the selective occurrence of recombination or hypermutation in different species and B-cell environments. Based on the finding that certain *AID* mutations affect switch recombination but not somatic hypermutation, it was suggested that AID needs the binding of a cofactor to start switch recombination (Barreto et al. 2003; Ta et al. 2003).

Analysis of knockout mutants of the chicken B-cell line DT40 indicate that the *RAD54* gene (Bezzubova et al. 1997) and other members of the RAD52 recombination repair pathway are needed for efficient Ig gene conversion (Sale et al. 2001). Most interestingly, disruption of *RAD51* paralogs reduces Ig gene conversion and induces hypermutation in the rearranged light chain gene (Sale et al. 2001), suggesting that a defect in DNA repair by homologous recombination can shift Ig gene conversion to hypermutation.

Valuable insight into complex recombination processes has been gained by the genetic and biochemical analysis of reaction intermediates (Haber 1998). Since sequence information needs to be copied from the donor to the target at some stage of Ig gene conversion, we reasoned that the deletion of the donor sequences might arrest the reaction and allow the recovery of an intermediate.

Here we report that ablation of pseudo V (ψV) donors activates AID-dependent Ig hypermutation in DT40 cells. This shows that Ig gene conversion and hypermutation are competing pathways derived from the same AID-initiated intermediate. Furthermore we propose ψV knockout DT40 as an ideal model system to approach the molecular mechanism of Ig hypermutation and as a new tool for in situ mutagenesis.

## Results

### Targeted Deletion of ψV Donor Sequences in the Rearranged Light Chain Locus

**Figure 1:**
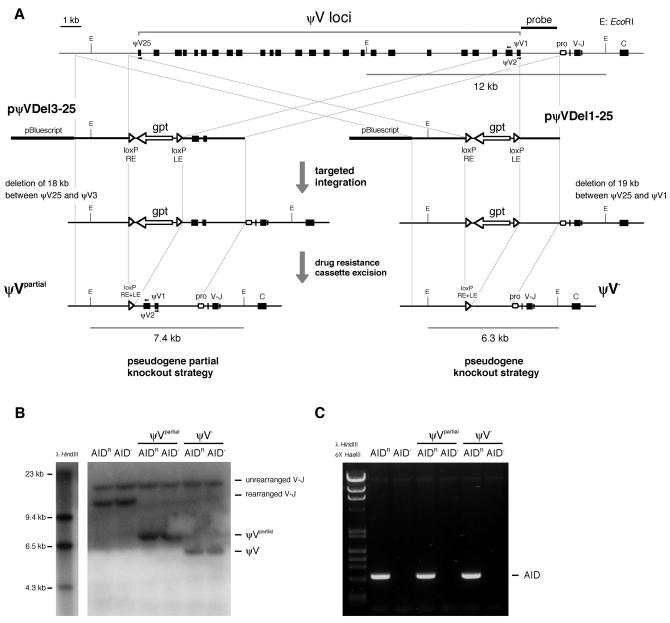
ψV Gene Deletion (A) Physical map of the rearranged Ig light chain locus in the chicken B-cell line DT40 and the ψV knockout constructs. The locus contains a total of 25 ψV genes upstream of the functional V segment. The strategy of knocking out ψV genes by the targeted integration of the pψVDel1-25 and the pψVDel3-25 constructs is shown. Only the relevant EcoRI sites are indicated. (B) Southern blot analysis of wild-type and knockout clones using the probe shown in (A) after EcoRI digestion. The wild-type locus hybridizes as a 12-kb fragment, whereas ψV^partial^ and ψV^–^ loci hybridize as 7.4-kb and 6.3-kb fragments, respectively. (C) AID status. The *AID* gene was amplified by PCR to verify the presence or absence of the *AID* cDNA expression cassette.

Two ψV knockout constructs were made by cloning genomic sequences that flank the intended deletion end points, upstream and downstream of a floxed *guanine phosphoribosyl transferase (gpt)* cassette (Arakawa et al. 2001). Upon targeted integration, the first construct, pψVDel1-25, deletes all pseudogenes (ψV25 to ψV1), whereas the second construct, pψVDel3-25, deletes most pseudogenes (ψV25 to ψV3) (Figure 1A). A surface IgM–positive (sIgM[+]) clone, derived from DT40^Cre1^AID^–/–^ cells (Arakawa et al. 2002) by transfection and stable integration of a floxed *AID–internal ribosome entry site (IRES)-green fluorescent protein (GFP)* transgene, was chosen for the transfection of the ψV knockout constructs. This AID-reconstituted clone, named AID^R^, has the advantage that the appearance of deleterious Ig light chain mutations can be easily detected by the loss of sIgM expression, and that GFP-marked *AID* expression can be shut down after tamoxifen induction of the *Cre* recombinase transgene inherited from DT40^Cre1^ (Arakawa et al. 2002).

Following transfection of the ψV knockout constructs into the AID^R^ clone, mycophenolic acid–resistant clones containing targeted deletions of the rearranged light chain locus were identified. These primary ψV knockout clones contain two floxed transgenes, the inserted *gpt* marker gene in the rearranged light chain locus and the *AID-IRES-GFP* gene of the AID^R^ progenitor clone. Since the *gpt* gene might perturb the adjacent transcription or chromatin configuration, the primary ψV knockouts were exposed to a low concentration of tamoxifen and then subcloned by limited dilution. In this way, secondary ψV knockout clones could be isolated that lacked either only the *gpt* gene (AID^R^ψV^–^ and AID^R^ψV^partial^) or the *gpt* gene together with the *AID-IRES-GFP* gene (AID^–/–^ψV^–^ and AID^–/–^ψV^partial^). The disruption of ψV genes in the rearranged light chain locus and the excision of the AID overexpression cassette were confirmed by Southern blot analysis (Figure 1B) and PCR (Figure 1C), respectively.

### Increased Loss of sIgM Expression after Deletion of ψV Genes in AID-Positive Clones

**Figure 2:**
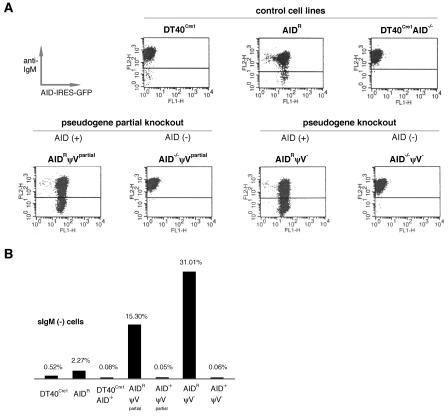
sIgM Expression Analysis of Control and ψV Knockout Clones (A) FACS anti-IgM staining profiles of representative subclones derived from initially sIgM(+) clones. (B) Average percentages of events falling into sIgM(–) gates based on the measurement of 24 subclones.

To estimate the rates of deleterious Ig mutations, sIg expression was measured by fluorescence-activated cell sorting (FACS) after 2 weeks' culture for 24 subclones each of the DT40^Cre1^, AID^R^, DT40^Cre1^AID^–/–^, and ψV knockout clones (Figure 2). Analysis of the controls with the intact ψV locus revealed an average of 0.52% and 2.27% sIgM(–) cells for the DT40^Cre1^ and AID^R^ subclones respectively, but only 0.08% for the DT40^Cre1^AID^–/–^. Previous analysis of spontaneously arising sIgM(–) DT40 variants demonstrated that about a third contained frameshift mutations in the rearranged light chain V segment that were regarded as byproducts of the Ig gene conversion activity (Buerstedde et al. 1990). This view is now supported by the finding that the AID-negative DT40^Cre1^AID^–/–^ clone, which should have lost the Ig gene conversion activity, stably remains sIgM(+). Most interestingly, subclones of the AID-positive ψV knockout clones (AID^R^ψV^partial^ and AID^R^ψV^–^) rapidly accumulate sIgM(–) populations, whereas subclones of the AID-negative ψV knockout clones (AID^–/–^ψV^partial^ and AID^–/–^ψV^–^) remain sIgM(+) (Figure 2). This suggests that the deletion of the pseudogenes dramatically increases the rate of deleterious light chain mutations in AID-expressing cells.

### Replacement of Ig Gene Conversion by Hypermutation in the Absence of ψV Donors

**Figure 3:**
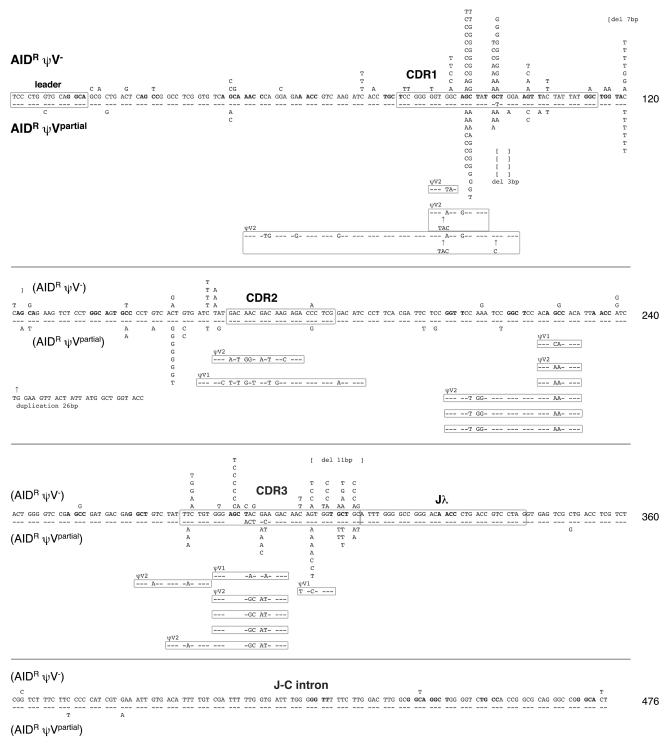
Ig Light Chain Sequence Analysis of the ψV Knockout Clones Mutation profiles of the AID^R^ψV^–^ and AID^R^ψV^partial^ clones. All differences identified in different sequences in the region from the leader sequence to the J-C intron are mapped onto the rearranged light chain sequence present in the AID^R^ precursor clone. Mutations of the AID^R^ψV^–^ and AID^R^ψV^partial^ clones are shown above and below the reference sequence, respectively. Deletions, insertions, and gene conversion events are also indicated. Hotspot motifs (RGYW and its complement WRCY) are highlighted by bold letters. Changes displayed in the same horizontal line are not necessarily derived from the same sequence.

**Figure 4:**
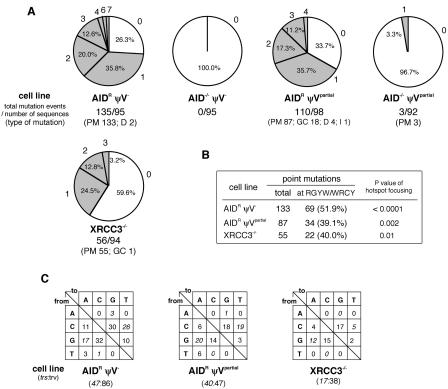
Mutation Profiles of Hypermutating Cell Lines (A) Percentages of sequences carrying a certain number of mutations. Each untemplated nucleotide substitution is counted, but gene conversions, deletions, and insertions involving multiple nucleotides are counted as single events. PM, point mutation; GC, gene conversion; D, deletion; I, insertion. (B) Hotspot preferences of untemplated nucleotide substitution mutations. Mutations occurring within a hotspot motif (RGYW or its complement WRCY) are shown by percentages. The hotspot preference was statistically significant (*p* < 0.05) by the standard difference test. (C) Patterns of nucleotide substitutions within sequences from ψV and the XRCC3 knockout clones. Nucleotide substitutions as part of gene conversion events are excluded. The ratios of transitions (trs) to transversions (trv) are also shown.

To analyze the newly identified mutation activity, the rearranged light chain VJ segments of the ψV knockout clones were sequenced 5–6 weeks after subcloning. A total of 135 nucleotide changes were found in the 0.5-kb region between the V leader and the 5′ end of the J-C intron within 95 sequences from the AID^R^ψV^–^ clone (Figure 3, upper reference sequence). In contrast to the conversion tracts seen in wild-type DT40 cells, almost all changes are single base substitutions, and, apart from a few short deletions and dinucleotide changes, mutation clusters were not observed. The lack of conversion events in AID^R^ψV^–^, which still contains the ψV genes of the unrearranged light chain locus, confirms that Ig gene conversion recruits only the ψV genes on the same chromosome for the diversification of the rearranged light chain gene (Carlson et al. 1990). No sequence diversity was found in a collection of 95 light chain gene sequences from the AID^–/–^ψV^–^ clone (Figure 4A; Table 1), indicating that AID is required for the mutation activity.

**Table 1:**
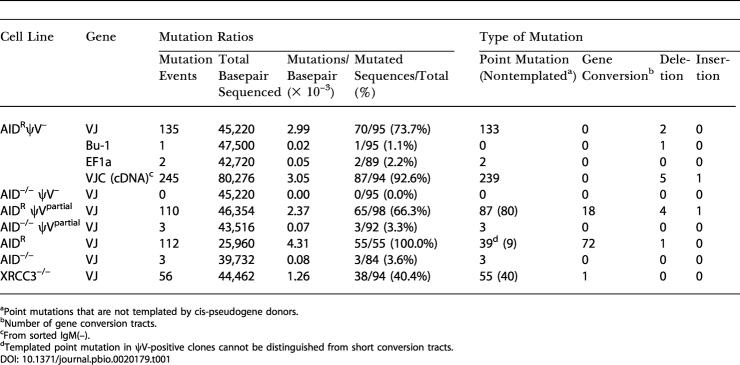
Mutation Profile

^a^Point mutations that are not templated by cis-pseudogene donors

^b^Number of gene conversion tracts

^c^From sorted IgM(–)

^d^Templated point mutation in ψV-positive clones cannot be distinguished from short conversion tracts

Sequences derived from the AID^R^ψV^partial^ clone occasionally display stretches of mutations that can be accounted for by the remaining ψV1 and ψV2 (Figure 3, lower reference sequence). Nevertheless, the majority of AID^R^ψV^partial^ mutations are single untemplated base substitutions as seen with the AID^R^ψV^–^ cells (Figure 4A; Table 1). Only three base substitutions, which are possibly PCR artifacts, were found in 92 sequences of the AID^–/–^ψV^partial^ clone, confirming that both the gene conversion and the mutation activities of AID^R^ψV^partial^ are AID-dependent.

### The New Mutation Activity of the ψV Knockout Clones Closely Resembles Somatic Hypermutation

The Ig mutation activity discovered in the ψV knockout clones with a predominance of single nucleotide substitutions suggests that somatic hypermutation had replaced Ig gene conversion. There is, however, a difference between the nucleotide substitutions in the AID^R^ψV^partial^ and AID^R^ψV^–^ clones and Ig hypermutations in germinal center B-cells: The clones show very few mutations in A/T bases and a preference for transversion mutations, and among transversions, a preference for G-to-C and C-to-G changes (Figure 4).

**Figure 5:**
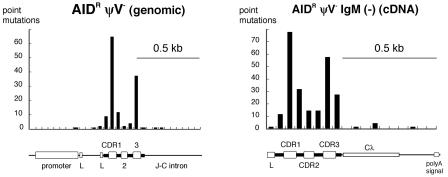
Mutations within Unsorted and Sorted sIgM(–) cells Distribution of nucleotide substitutions within genomic sequences from unsorted AID^R^ψV^–^ cells and within cDNA sequences from sorted IgM(–) AID^R^ψV^–^ cells. The numbers of mutations are counted for every 50 bp, and are shown together with the corresponding physical maps of the light chain genomic locus or the cDNA sequence.

Ig hypermutations are typically localized within 1 kb of the transcribed gene sequence, with preferences for the complementary determining regions (CDRs) of the V(D)J segments, whereas no or few mutations are present in the downstream C region (Lebecque and Gearhart 1990). To investigate whether the mutations in the AID^R^ψV^–^ clone follow a similar distribution, sequence analysis was extended to the promoter region and the J-C intron of the rearranged light chain gene (Figure 5). Although mutations are found close to the promoter and in the intron downstream of the J segments, the peak incidence clearly coincides with the CDR1 and CDR3, which are also preferred sites of gene conversion in DT40 (unpublished data). Approximately half of all point mutations fall within the RGYW (R = A/G; Y = C/T; W = A/T) sequence motif or its complement WRCY (see Figure 4B), known as hotspots of Ig hypermutation in humans and mice.

It was previously reported that the deletion of *RAD51* paralogs induces Ig hypermutation in DT40 cells (Sale et al. 2001). To compare the hypermutation activity in the ψV gene-negative and *RAD51* paralog-negative backgrounds, the *XRCC3* gene was disrupted in the DT40^Cre1^ clone, and the rearranged VJ genes were sequenced 6 weeks after subcloning. The mutation spectrum of the *XRCC3-*deficient clone was similar to that of the AID^R^ψV^–^ clone (see Figure 4C) and to what was previously reported for the *XRCC3* knockout (Sale et al. 2001). Nevertheless, the mutation rate in the new *XRCC3* mutant was about 2.5-fold lower than in the AID^R^ψV^–^ clone, and there was a clear slow-growth phenotype of the *XRCC3* mutant compared to wild-type DT40 and the AID^R^ψV^–^ clone (unpublished data).

To identify the mutations responsible for the loss of sIgM expression in the AID^R^ψV^–^ clone, 94 light chain cDNAs from sorted sIgM(–) cells were amplified and sequenced. Although one short insertion and five deletions were detected in this collection (Table 1), 89% of the 245 total mutations are single-nucleotide substitutions within the VJ segments and only few mutations were observed in the C segment (Figure 5). Surprisingly, only about 10% of the sequences contained a stop codon or a frameshift, suggesting that the lack of sIgM expression is mainly caused by amino acid substitutions that affect the pairing of the Ig light and heavy chain proteins.

### Ig Locus Specificity of Hypermutation

It has been reported that high AID expression in fibroblasts (Yoshikawa et al. 2002) and B-cell hybridomas (Martin and Scharff 2002) leads to frequent mutations in transfected transgenes. To rule out the possibility that the pseudogene deletions had induced a global hypermutator phenotype, the 5′ ends of the genes encoding the B-cell -specific marker *Bu-1* and the translation elongation factor *EF1α* were sequenced for the AID^R^ψV^–^ clone. Only a single 1-bp deletion was found within 95 sequences of the *Bu-1* gene, and only two single nucleotide substitutions within 89 sequences of *EF1α* (Table 1). As these changes most likely represent PCR artifacts, this further supports the view that the hypermutations induced by the ψV deletions are Ig-locus-specific.

## **Discussion**

These results demonstrate that the deletion of the nearby pseudogene donors abolishes Ig gene conversion in DT40 and activates a mutation activity that closely resembles Ig hypermutation. The features shared between this new mutation activity and somatic hypermutation include (1) AID dependence, (2) a predominance of single nucleotide substitutions, (3) distribution of the mutations within the 5′ transcribed region, (4) a preference for hotspots, and (5) Ig gene specificity. The only differences between the mutation activity induced by loss of ψV and Ig hypermutation in vivo are the relative lack of mutations in A/T bases and a predominance of transversion mutations in the ψV knockout clones. However, these differences are also seen in hypermutating Epstein Barr virus–transformed B-cell lines (Bachl and Wabl 1996; Faili et al. 2002) and DT40 mutants of *RAD51* paralogs (Sale et al. 2001), indicating that part of the Ig hypermutator activity is missing in transformed B-cell lines. Interestingly, the rate of Ig hypermutation in the AID^R^ψV^–^ clone seems higher than the rate of Ig gene conversion in the DT40^Cre1^ progenitor. An explanation for this could be that some conversion tracts are limited to stretches of identical donor and target sequences and thus leave no trace. The ratio of transversion to transition was lower for the AID^R^ψV^partial^ clone (see Figure 4). Although we can only speculate about the cause of this difference, it might be due to the correction of point mutations by mismatch correction of one or more sites in gene conversion tracts.

**Figure 6:**
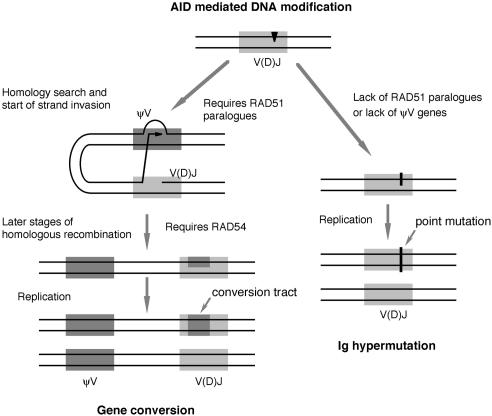
A Model of the Regulation of Ig Gene Conversion and Hypermutation

The induction of Ig hypermutation by the blockage of Ig gene conversions supports a simple model explaining how hypermutation and recombination are initiated and regulated (Figure 6). Initiating the events is a modification of the rearranged V(D)J segment that is either directly or indirectly induced by AID. The default processing of this lesion in the absence of nearby donors or of high homologous recombination activity leads to Ig hypermutation in the form of a single nucleotide substitution (Figure 6, right). However, if donor sequences are available, processing of the AID-induced lesion can be divided into a stage before strand exchange, when a shift to Ig hypermutation is still possible, and a stage after strand exchange, when the commitment toward Ig gene conversion has been made (Figure 6, left). Whereas completion of the first stage requires the participation of the *RAD51* paralogs, the second stage involves other recombination factors, such as RAD54.

This difference in commitment explains why disruptions of the *RAD51* paralogs not only decrease Ig gene conversion, but also induce Ig hypermutation (Sale et al. 2001), whereas disruption of the *RAD54* gene only decreases Ig gene conversion (Bezzubova et al. 1997). The model also predicts that low cellular homologous recombination activity prevents Ig gene conversion even in the presence of conversion donors. Such a low homologous recombination activity might be the reason why human and murine B-cells never use Ig gene conversion despite the presence of nearby candidate donors in the form of unrearranged V segments and why chicken germinal center B-cells have shifted the balance from Ig gene conversion to Ig hypermutation (Arakawa et al. 1998).

The AID^R^ and the ψV knockout DT40 clones are a powerful experimental system to address the role of trans-acting factors and cis-acting regulatory sequences for Ig gene conversion and hypermutation. Compared to alternative animal or cell culture systems, it offers the advantages of (1) parallel analysis of Ig gene conversion and hypermutation, (2) conditional AID expression, (3) easy genome modifications by gene targeting, (4) normal cell proliferation and repair proficiency, and (5) Ig locus specificity of hypermutation. The ability to induce gene-specific hypermutation in the DT40 cell line might also find applications in biotechnology. One possibility is to replace the chicken antibody coding regions with their human counterparts and then to simulate antibody affinity maturation from a repertoire that continuously evolves by Ig hypermutation.

## Materials and Methods

### 

#### Cell lines

DT40^Cre1^, which displays increased Ig gene conversion due to a v-*myb* transgene and contains a tamoxifen-inducible Cre recombinase, has been described previously (Arakawa et al. 2001). DT40^Cre1^AID^–/–^ was generated by the targeted disruption of both *AID* alleles of DT40^Cre1^ (Arakawa et al. 2002). AID^R^ was derived from DT40^Cre1^AID^–/–^ after stable integration of a floxed *AID-IRES-GFP* bicistronic cassette, in which both *AID* and *GFP* are expressed from the same β-actin promoter. AID^R^ψV^–^ was derived from AID^R^ by transfection of pψVDel1-25 (see Figure 1A). Stable transfectants that had integrated the construct into the rearranged light chain locus were then identified by locus-specific PCR. Targeted integration of pψVDel1-25 results in the deletion of the entire ψV gene loci starting 0.4 kb upstream of ψV25 and ending 1 bp downstream of ψV1. AID^R^ψV^partial^ was produced in a similar way as was AID^R^ψV^–^, by transfection of pψVDel3-25, which, upon targeted integration, leads to a partial deletion of the ψV loci starting 0.4 kb upstream of ψV25 and ending 1 bp downstream of ψV3. Cell culture and electroporation were performed as previously described (Arakawa et al. 2002). XRCC3^–/–^ was derived from DT40^Cre1^ by deleting amino acids 72–170 of the *XRCC3* gene following transfection of XRCC3 knockout constructs. Clones that underwent targeted integration were initially identified by long-range PCR, and the *XRCC3* deletion was then confirmed by Southern blot analysis.

#### Ig reversion assay

Subcloning, antibody staining, flow cytometry, and quantification of sIgM expression has been described previously (Arakawa et al. 2002). All clones used in the study were sIgM(+) because of the repair of the light chain frameshift of the original Cl18(–) variant (Buerstedde et al. 1990) by a gene conversion event.

#### **PCR**

To minimize PCR-introduced artificial mutations, PfuUltra hotstart polymerase (Stratagene, La Jolla, California, United States) was used for amplification prior to sequencing. Long-range PCR, RT-PCR, and Ig light chain sequencing were performed as previously described (Arakawa et al. 2002). The promoter and J-C intron region of Ig light chain plasmid clones were sequenced using the M13 forward and reverse primers. *Bu-1* and *EF1α* genes were amplified using BU1/BU2 (BU1, 5′-GGGAAGCTTGATCATTTCCTGAATGCTATATTCA-3′; BU2, 5′-GGGTCTAGAAACTCCTAGGGGAAACTTTGCTGAG-3′) and EF6/EF8 (EF6, 5′-GGGAAGCTTCGGAAGAAAGAAGCTAAAGACCATC-3′; EF8, 5′-GGGGCTAGCAGAAGAGCGTGCTCACGGGTCTGCC-3′) primer pairs, respectively. The PCR products of these genes were cloned into the pBluescript plasmid vector (Stratagene) and were sequenced using the M13 reverse primer.

## Supporting Information

### Accession Numbers

The GenBank (http://www.ncbi.nlm.nih.gov/) accession numbers of the genes discussed in this paper are as follows. *AID* (NM_009645; NM_020661), *RAD54* (GGU92461), *RAD52* (U01047), *Bu-1* (X92865), and *EF1α* (NM_204157).

## Abbreviations

ψV: pseudo V
AID: activation-induced cytidine deaminase
CDR: complementary determining region
FACS: fluorescence-activated cell sorting
GFP: green fluorescent protein
gpt: guanine phosphoribosyl transferase
Ig: immunoglobulin
IRES: internal ribosome entry site
sIg: surface Ig
